# Editorial: Genetically deciphering the adaptive growth and development of photosynthetic organisms: a holistic omics approach

**DOI:** 10.3389/fpls.2024.1474114

**Published:** 2024-09-13

**Authors:** Dongli He, Maoteng Li, Jiangxin Wang, Xin Wang

**Affiliations:** ^1^ State Key Laboratory of Biocatalysis and Enzyme Engineering, School of Life Sciences, Hubei University, Wuhan, China; ^2^ Department of Biotechnology, College of Life Science and Technology, Huazhong University of Science and Technology, Wuhan, China; ^3^ Shenzhen Key Laboratory of Marine Bioresource and Eco-Environmental Science, Shenzhen Engineering Laboratory for Marine Algal Biotechnology, Shenzhen, China; ^4^ Guangdong Provincial Key Laboratory for Plant Epigenetics, College of Life Sciences and Oceanography, Shenzhen University, Shenzhen, China; ^5^ Key Laboratory of Biology and Genetic Improvement of Oil Crops, Ministry of Agriculture and Rural Affairs, Oil Crops Research Institute of the Chinese Academy of Agricultural Sciences, Wuhan, China

**Keywords:** plant, adaptation, development, genomics, transcriptomics, proteomics, metabolomics

## Introduction

In recent years, the field of plant biology has significantly benefited from advancements in omics technologies, enabling a comprehensive understanding of the adaptive growth and development of photosynthetic organisms. These technologies, which encompass genomics, transcriptomics, proteomics, metabolomics, and more, provide a holistic view of the complex biological processes underlying plant adaptation and development. This special topic editorial highlights recent research that leverages these omics approaches to decode the genetic basis of plant adaptation, emphasizing the integration of multi-omics data to uncover new insights and potential applications in crop improvement and environmental sustainability.

## Genomics

The publication of the *Arabidopsis* genome in 2000 marked a milestone in plant genomics, paving the way for the sequencing of over 700 plant genomes. These efforts have propelled population genetics and crop breeding by facilitating large-scale resequencing projects, such as next-generation sequencing (NGS) and long-read sequencing (the third generation, Pacific Bioscience and Oxford Nanopore technology). For instance, the domestication patterns of lotus (*Nelumbo nucifera* Gaertn.) were explored using resequencing data from 371 lotus accessions, revealing subgroup-specific metabolic pathways in domesticated regions (Qi et al.). This genomic information is crucial for identifying breeding genes related to key agronomic traits such as plant architecture and flowering time (Varshney et al., 2017; Liu et al., 2020; Kress et al., 2022).

## Transcriptomics

Transcriptomic analyses, using technologies like RNA sequencing (RNA-seq), have advanced our understanding of how photosynthetic organisms reprogram gene expression in response to environmental stresses. For example, a pan-transcriptome analysis of rice cultivars under cold treatment identified key genes involved in cold tolerance, highlighting the role of alternative splicing in stress responses (Zhong et al., 2024). Similarly, full-length transcriptome sequencing of peanut cultivars with contrasting cold tolerance provided insights into the significance of alternative splicing in enhancing cold resistance (Wang et al.). In longan (*Dimocarpus longan* Lour.), comparative transcriptome analysis identified genes related to sucrose accumulation during fruit maturation, which are vital for improving fruit quality (Li et al.). The transcriptional regulations by HFR1 and HY5 in response to shade in *Arabidopsis* were explored, revealing the complex interplay among these factors in coordinating shade avoidance responses (Choi et al.).

## Proteomics

Proteomics has made significant strides with the development of high-resolution mass spectrometry and associated bioinformatics tools, such as iTRAQ, data-dependent acquisition (DDA), and data-independent acquisition (DIA/SWATH). These advancements allow for the detailed characterization of protein expression, localization, interactions, and modifications. For example, a proteomic study on *Arabidopsis thaliana* created a high-throughput expression atlas of 10 organs, covering 15,514 protein groups, which is approximately 56.5% of the predicted proteome (Zhang et al., 2019). Such studies are instrumental in understanding protein dynamics during plant development and stress responses. In barley, a genome-wide analysis of the TALE gene family identified 21 HvTALE genes responsive to exogenous hormones, providing insights into their regulatory roles (Liao et al.).

## Metabolomics

Metabolomics offers a high-throughput and quantitative approach to studying the vast array of metabolites in plants, aiding in the understanding of metabolic regulation and adaptation. Technologies like GC-MS, LC-MS, and NMR are commonly used to analyze plant metabolites. Integrating metabolomics with other omics data, such as genomics and proteomics, helps decipher the complex regulatory networks in plants. For instance, through the integration of metabolomics and transcriptomics analyses, the dynamic accumulation of major secondary metabolites was investigated in the maturing seed plumule of the sacred lotus and several systematic metabolic regulation maps were established accordingly (He et al., 2023). A combined metabolomic and proteomic analysis of *Sesuvium portulacastrum* under salt stress illustrated the integrative mechanisms of salt tolerance, providing a working model for its adaptive responses (Cao et al.).

## Conclusion and perspective

The integration of multi-omics approaches offers a powerful strategy to unravel the genetic and molecular mechanisms underlying the adaptive growth and development of photosynthetic organisms. By combining data from genomics, transcriptomics, proteomics, and metabolomics, researchers can gain a comprehensive understanding of plant biology, laying the foundation for innovative applications in agriculture and environmental management. The six papers collected on this special topic provide a comprehensive exploration of the adaptive growth and development of photosynthetic organisms through various omics approaches, illustrating the immense potential of omics technologies to decode the genetic basis of plant adaptation. From exploring the intricate mechanisms of salt tolerance in halophytes to uncovering the genetic underpinnings of cold resistance in peanuts and sucrose accumulation in longan, these studies highlight the diverse strategies for plants to thrive under various environmental stresses.

Moving forward, a holistic omics approach will be essential to gain a comprehensive understanding of how photosynthetic organisms respond to and cope with changing environments. By integrating data across different omics layers, we can build detailed models of plant adaptation, identify key regulatory genes and pathways, and develop innovative strategies for crop improvement. Such knowledge will be pivotal in enhancing the resilience and productivity of crops, ensuring food security, and promoting sustainable agricultural practices in the face of global climate change.

In conclusion, the future of plant biology largely lies in the seamless integration of multi-omics technologies. By genetically deciphering the adaptive growth and development of photosynthetic organisms, we can unlock new potentials for crop improvement and environmental sustainability, paving the way for a greener and more resilient world.
